# Insight into Seasonal Change of Phytochemicals, Antioxidant, and Anti-Aging Activities of Root Bark of *Paeonia suffruticosa* (Cortex Moutan) Combined with Multivariate Statistical Analysis

**DOI:** 10.3390/molecules26206102

**Published:** 2021-10-10

**Authors:** Shicong Yang, Xiaoyan Liu, Jingyu He, Menghua Liu

**Affiliations:** 1 Zhongshan School of Medicine, Sun Yat-Sen University, Guangzhou 510080, China; chenliangyj@sina.com; 2 NMPA Key Laboratory for Research and Evaluation of Drug Metabolism, Guangdong Provincial Key Laboratory of New Drug Screening, School of Pharmaceutical Sciences, Southern Medical University, Guangzhou 510515, China; liu1791552373@163.com; 3 Bioengineering Research Centre, Guangzhou Institute of Advanced Technology, Chinese Academy of Sciences, Guangzhou 511458, China

**Keywords:** Cortex Moutan, composition, antioxidant, anti-aging, collection period, multivariate statistical analysis

## Abstract

Chemical compositions, antioxidants, and anti-aging activities of Cortex Moutan (CM), from different collection periods and different producing areas, were measured and compared in order to obtain excellent CM extracts. The bioactivities of CM extracts were examined by an in vitro antioxidant method and a UVB irradiated human dermal fibroblast (HDF) model. Phytochemical properties were obtained from ultra-fast liquid chromatography quadrupole time-of-flight mass spectrometry (UFLC-Q-TOF-MS) prior to the multivariate statistical analysis. As for the results, the extracts of Heze CM (HZCM) and Luoyang CM (LYCM) collected in June had better in vitro antioxidant activities, significantly increased the activities of superoxide dismutase (SOD) and glutathione peroxidase (GSH-Px), and reduced the content of malondialdehyde (MDA), compared to other CM extracts. HZCM and LYCM extracts could upregulate the relative expression of SOD and GSH-Px mRNA. The extract of HZCM collected in June could significantly repress the production of matrix metalloproteinase 1 (MMP-1) and improve the production of procollagen type I (PCOL)-I in UVB irradiated HDF. In total, 50 compounds, including 17 monoterpenoids, 19 flavonoids, 13 phenols, and 1 amino acid were identified or tentatively identified in the CM extracts. Gallic acid, *p*-hydroxybenzoic acid, oxypaeoniflorin, paeoniflorin, 1,2,3,4,6-*O*-pentagalloyl glucose, and paeonol were predominant compounds in the CM extracts. Taken together, CM collected from April to September had better antioxidant and anti-aging effects for external usage.

## 1. Introduction

*Paeonia suffruticosa*, belonging to Paeoniaceae, is mainly distributed in China, Japan, America, and Europe [1,2]. It is an ornamental, edible and medicinal plant in China [3]. Cortex Moutan (CM), root bark of *P*. *suffruticosa*, is an important Chinese traditional medicine with anti-inflammatory, anti-bacterial, and anti-tumor effects; it lowers blood pressure, lowers blood sugar, and regulates the cardiovascular system [4]. Phytochemical research has shown that phenols and their glycosides, monoterpenes, glycosides, flavonoids, polysaccharides, organic acids, amino acids, and volatile oil are isolated and identified in CM [4,5].

In recent years, CM has been used for external usage, especially skin problems, mainly focusing on antioxidant, anti-aging, whitening, antibacterial, and other aspects, which is different from oral administration [6,7,8,9,10]. Traditionally, the content of paeonol is an important factor in evaluating the quality of CM, according to Chinese Pharmacopoeia [11]. It is reported that the content of paeonol in CM collected from September to October was the highest; commercially, the superior CM is usually collected in this period [12,13]. As known, the biological efficacy of plant medicine is the result of the overall regulation of a series of phytochemicals. A large number of phytochemicals that are responsible for the bioactivity are affected by the different growth periods [14,15]. It has been found that the optimal time to harvest the *P. suffruticosa* flower is before the early flowering stage, due to the excellent antioxidant and anti-photoaging activities and the high levels of bioactive phytochemicals. However, for external usage of CM, there is no principle guided for which the growth period is suitable for harvesting and no report on the quality standard of CM for external antioxidant and aging effects. The study of the relationship between phytochemicals and antioxidants or anti-aging activity during the growth period is also less documented, which limits the development of products originating from CM.

To date, ultra-performance liquid chromatography (UPLC) coupled with high-resolution mass spectrometry (HRMS) or quadrupole time-of-flight mass spectrometry (Q-TOF-MS) can be used successfully to analyze in the bioactive compounds in the different parts of herbal plants and the whole herb [16,17]. Multivariate statistical analysis, as an effective analytical tool, is also combined with the instrument to obtain useful information and to simplify the results. The differences in metabolites, critical compounds, and biomarkers in botanical origin, temporal and spatial distribution, growth period, and clinical use were elucidated based on the combined application of qualitative and quantitative results and multivariate statistical analysis [18,19,20].

In this study, the in vitro antioxidant activities of the ethanolic extracts of CM were measured using four different methods, including 2,2-diphenyl-1-picrylhydrazyl (DPPH) and hydroxyl radical scavenging activity, ferric reducing antioxidant power (FRAP), and inhibition of β-carotene bleaching assays. The ex vivo antioxidant and anti-aging activities of the ethanolic extracts of CM were measured using the UVB irradiated human dermal fibroblast (HDF) model. The characteristic compositions of the ethanolic extracts of CM were investigated by UFLC-Q-TOF-MS prior to the correlation analysis between the multiple components and their bioactivities.

## 2. Results and Discussion

### 2.1. Total Phenolic Content

The 50% ethanolic extract yields of eight CM samples had no obvious difference between 20.33% and 22.53% (Table 1). TPCs in these CM extracts were measured. TPC of HZCM-2 was the highest, followed by that of LYCM-6, both of which were collected in June. Among the other CM samples, TPCs of HZCM-3 and LYCM-3 collected in September were close, which were 80.80 ± 4.48 mg GAE/g extract and 80.70 ± 2.95 mg GAE/g extract, respectively. TPCs of HZCM-1 and LYCM-1 collected in March were 73.47 ± 1.01 mg GAE/g extract and 67.71 ± 2.09 mg GAE/g extract, respectively. However, they were higher than those collected in December.

Total phenolic compounds are a large class of plant secondary metabolites that perform many physiological functions, and are involved in many important metabolic reactions. They are often regarded as growth regulators and interact with specific plant hormones or function alone to affect the activity of certain enzymes [21,22]. The accumulation of phenolic compounds after rooting might be regulated by dynamic changes in the endogenous hormone contents. The maturity stage shows a significant influence, not only on the content of total phenolic compounds, but also on the polyphenolic profile, while environmental conditions during harvesting may also impose a significant impact on the phenolic compounds [23,24]. After the flowering stage in March and April, TPCs of CM samples will have peak values. The HZCM and LYCM extracts showed the similar trend on the change of TPC. The finding suggested a translocation of phenolic compounds to the most biosynthetically active plant roots.

### 2.2. Antioxidant Activity In Vitro

**Figure 1:**
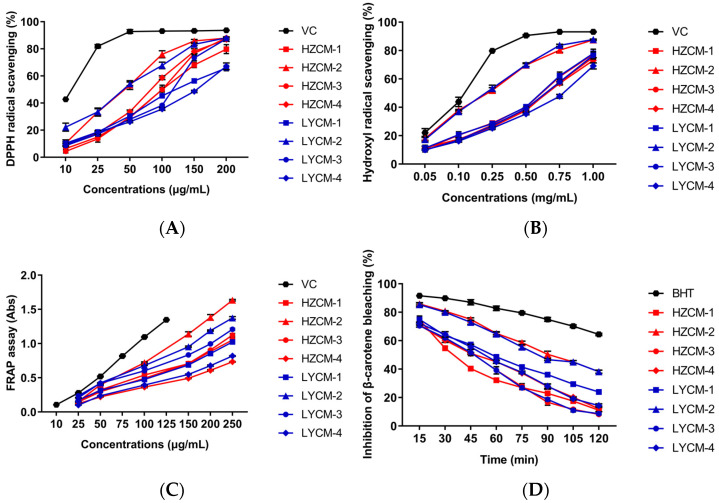
In vitro antioxidant effects of Cortex Moutan extracts. Effects of Cortex Moutan (CM) extracts on DPPH scavenging (**A**), hydroxyl radical scavenging (**B**), FRAP assay (**C**), and inhibition of β-carotene bleaching (**D**). Vitamin C: VC; HZCM-1, HZCM-2, HZCM-3, and HZCM-4: CM collected in Heze in March, June, September, and December, respectively. LYCM-1, LYCM-2, LYCM-3, and LYCM-4: CM collected in Luoyang in March, June, September, and December, respectively. Data were expressed as mean ± SD (*n* = 3).

The antioxidant properties of CM extracts were measured by the DPPH and hydroxyl radical scavenging, FRAP, and inhibition of β-carotene bleaching assays. The LYCM-2 extract had a stronger DPPH radical scavenging activity than other CM extracts, while it was obviously weaker than that of VC at concentration 10–200 μg/mL (Figure 1A). On the basic of IC_50_ values, antioxidant activity is defined as the concentration of antioxidant required for 50% scavenging of the radicals (Table 1). The IC_50_ values of LYCM-2 and HZCM-2 extracts for scavenging DPPH was 40.38 ± 1.86 μg/mL and 42.70 ± 0.79 μg/mL, respectively, displaying the close activity. The two IC_50_ values of LYCM-2 and HZCM-2 extracts were much lower than that of the others (Table 1). A lower IC_50_ value suggested antioxidant activity was better in the eight extracts. The LYCM-2 had a stronger hydroxyl radical scavenging activity than the other extracts, but weaker than VC. The IC_50_ value of HZCM-2 extract for scavenging hydroxyl radical was just a little higher than that of LYCM-2 extract, displaying strong scavenging activity. The extracts of HZCM-1 and LYCM-1 collected in March had very close activity and were stronger than that of the CM extracts collected in September and December (Figure 1B). In FRAP assay, HZCM-2 had the strongest activity, followed by LYCM-2 and LYCM-3. HZCM-1, HZCM-3, and LYCM-1 extracts had similar activity, which were much stronger than that of HZCM-4 and LYCM-4 collected in December (Figure 1C). The activities of HZCM-2 and LYCM-2 in the inhibition of the β-carotene bleaching assay was close, which were much weaker than that of BHT (100 μg/mL) over 120 min, but had stronger and lasted longer inhibitory activity than that of other CM extracts at 100 μg/mL (Figure 1D). Based on the results of the antioxidant evaluation, the antioxidant activities of HZCM-2 and LYCM-2 collected in June were noticeable, with a great effect on hydroxyl radical scavenging and inhibition of the β-carotene bleaching.

It is reported that a large number of phytochemicals could reduce free radicals and help to prevent against oxidation [25]. There are multiple free radical and oxidant sources found in the biosystem. In certain cases, individual antioxidants performed by the multiple mechanisms in a single system or by a different single mechanism, depending on the reaction system [26]. Moreover, antioxidants could respond in a different manner to different free radicals or oxidant sources [27]. In this study, the CM extracts, as a mixed and complex system, were evaluated by four different methods, which could reflect their antioxidant activities, comprehensively.

### 2.3. Effects of CM Extracts on Cell Viability, SOD, GSH-Px and MDA

**Figure 2:**
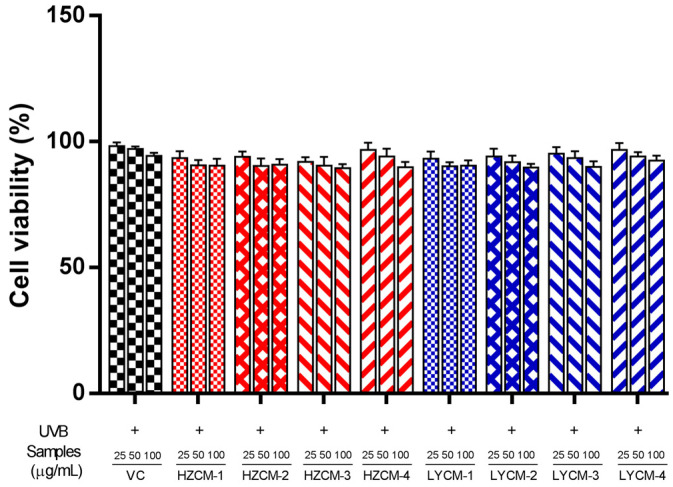
Effects of Cortex Moutan extracts on the viability of human dermal fibroblast. Vitamin C: VC; HZCM-1, HZCM-2, HZCM-3, and HZCM-4: Cortex Moutan (CM) collected in Heze in March, June, September and December, respectively. LYCM-1, LYCM-2, LYCM-3, and LYCM-4: CM collected in Luoyang in March, June, September, and December, respectively. Cell viability was studied at the three concentrations of VC and eight CM extracts (25, 50, and 100 µg/mL). Data are expressed as mean ± SD (*n* = 3).

As shown in Figure 2, the percentages of cell viability for the eight samples were from 91.83 to 97.62%. Cell viability was not significantly affected by the CM extracts at various concentrations (0–100 µg/mL) after UVB irradiation. These data indicated that these extracts had no toxic effect on cells at concentrations below 100 µg/mL. Subsequent experiments were performed at the concentrations of 25, 50 and 100 µg/mL.

**Figure 3:**
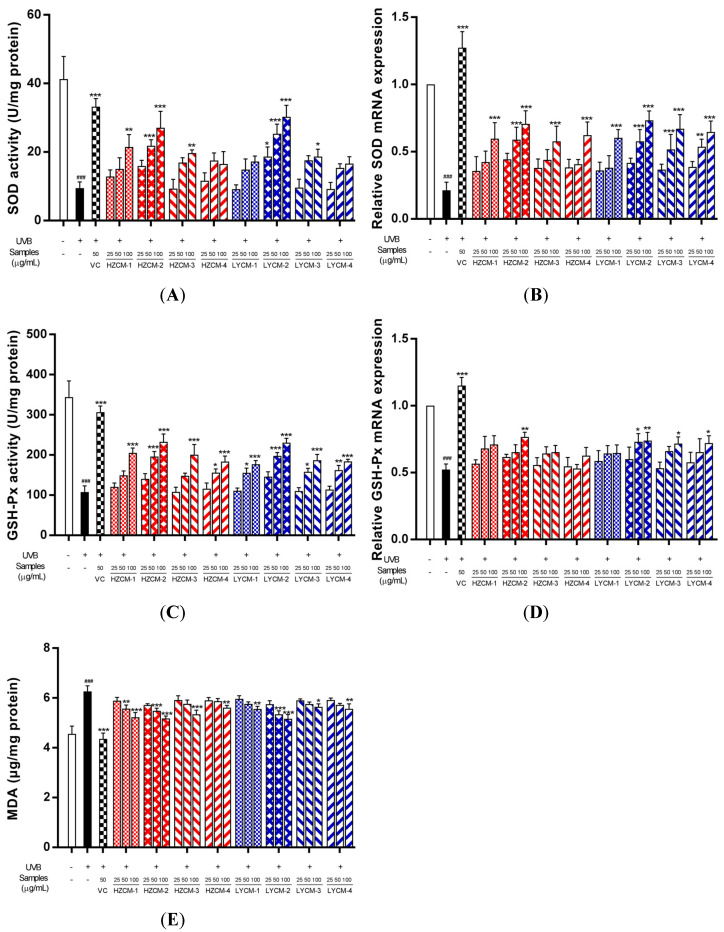
Ex vivo antioxidant effects of Cortex Moutan extracts. Effects of Cortex Moutan (CM) extracts on the activity of SOD (**A**), the relative expression of SOD mRNA (**B**), the activity of GSH-Px (**C**), the relative expression of GSH-Px mRNA (**D**), and the production of MDA (**E**). Vitamin C: VC; HZCM-1, HZCM-2, HZCM-3, and HZCM-4: Cortex Moutan collected in Heze in March, June, September, and December, respectively. LYCM-1, LYCM-2, LYCM-3, and LYCM-4: Cortex Moutan collected in Luoyang in March, June, September, and December, respectively. The content of malondialdehyde (MDA) and the activity of superoxide dismutase (SOD) and glutathione peroxidase (GSH-Px) were measured by colorimetric assay kits. SOD and GSH-Px mRNA expressions were examined by RT-PCR. The concentration of VC was 50 μg/mL, and three concentrations of eight CM extracts were 25, 50, and 100 μg/mL. Data are expressed as mean ± SD (*n* = 3). * *p* < 0.05, ** *p* < 0.01 and *** *p* < 0.001 versus the model group with UVB irradiation; ^###^ *p* < 0.001 versus the control group without UVB irradiation.

Compared with the control group, the activities of SOD and GSH-Px significantly decreased, and the content of MDA significantly increased when HDF was treated with UVB irradiation. However, when the HDF was treated with CM extracts, HZCM-1, HZCM-3, and LYCM-3 extracts at the concentration of 100 µg/mL could significantly increase the activity of SOD (Figure 3A). The activity of SOD was significantly increased by HZCM-2 extract at concentrations of 50 and 100 µg/mL and LYCM-2 extract at concentrations of 25, 50, and 100 µg/mL. The relative expression of SOD mRNA was significantly downregulated by HZCM-2, LYCM-2, LYCM-3, and LYCM-4 extracts at a concentration of 50 µg/mL, and significantly downregulated by all CM extracts at the concentrations of 100 µg/mL (Figure 3B). In addition to HZCM-1 and HZCM-3 extracts, other CM extracts significantly increased the activity of GSH-Px at a concentration of 50 µg/mL. All extracts significantly increased the activity of GSH-Px at the concentration of 100 µg/mL (Figure 3C). The relative expression of GSH-Px mRNA was upregulated by LYCM-2 extract at the concentration of 50 µg/mL and HZCM-2, LYCM-2, LYCM-3, and LYCM-4 extracts at the concentration of 100 µg/mL (Figure 3D). Only at the concentration of 100 µg/mL, HZCM-3, HZCM-4, LYCM-3, and LYCM-4 extracts could significantly reduce the content of MDA. HZCM-1, HZCM-2, LYCM-1, and LYCM-2 extracts could significantly reduce the content of MDA at the concentrations of 50 and 100 µg/mL (Figure 3E).

The free radicals of oxygen increase cell antioxidant capacity and oxidant production. They are not deleterious and play vital roles in cell signaling and defense when they are at a relatively low level. However, at a higher level, they are harmful to DNA and proteins [28]. The antioxidant enzymes, such as SOD and GSH-Px, play crucial roles in maintaining homeostasis into cells. SOD is to scavenge superoxide radicals and convert superoxide anion to hydrogen peroxide and molecular oxygen [29]. GSH-Px is achieved by the reduction of hydrogen peroxide, lipid hydroperoxides, and other organic hydroperoxides [30]. It was found that the enzymatic activities of SOD and GSH-Px in the cells treated with CM extracts were increased after UVB irradiation. MDA, the final product of lipoperoxidation, is a highly electrophilic molecule that reacts with cell nucleophiles to form MDA adducts and MDA oligomers. Moreover, MDA as a biomarker is the principal and most studied product of lipid peroxidation [31]. In this study, CM extracts could significantly reduce the content of MDA. These results suggest that the CM extracts could enhance the antioxidant defense mechanism in UVB irradiated cells.

### 2.4. Effects on MMP-1 and PCOL-I

**Figure 4:**
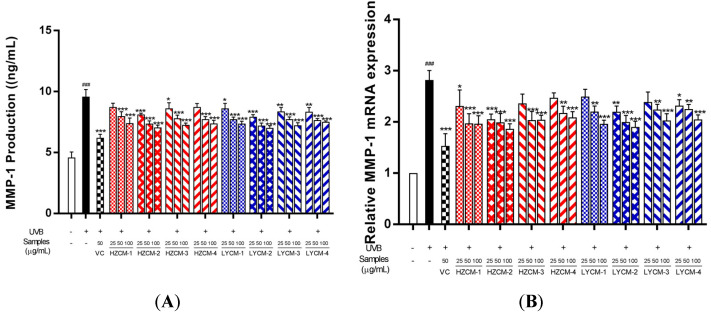

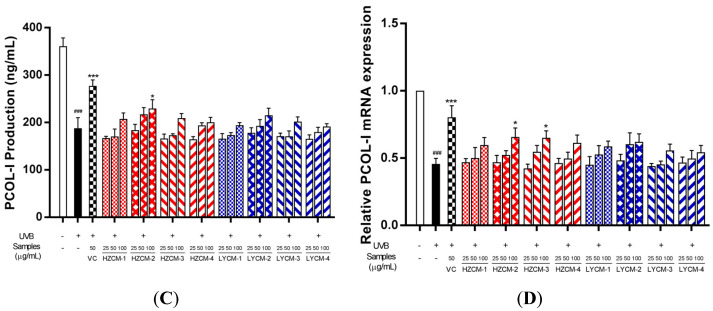
Ex vivo anti-aging effects of Cortex Moutan extracts. Effects of Cortex Moutan (CM) extracts on the production of MMP-1 (**A**), the relative expression of MMP-1 mRNA (**B**), the production of PCOL-I (**C**), the relative expression of PCOL-I mRNA (**D**). Vitamin C: VC; HZCM-1, HZCM-2, HZCM-3, and HZCM-4: CM collected in Heze in March, June, September, and December, respectively. LYCM-1, LYCM-2, LYCM-3, and LYCM-4: CM collected in Luoyang in March, June, September, and December, respectively. The levels of MMP-1 and PCOL-I were measured by enzyme-linked immunosorbent assay (ELISA) kits. MMP-1 and PCOL-I mRNA expressions were examined by RT-PCR. The concentration of VC was 50 μg/mL, and three concentrations of eight CM extracts were 25, 50 and 100 μg/mL. Data are expressed as mean ± SD (*n* = 3). * *p* < 0.05, ** *p* < 0.01 and *** *p* < 0.001 versus the model group with UV irradiation; ^###^ *p* < 0.001 versus the control group without UVB irradiation.

After UVB irradiation, the content of MMP-1 in the model group significantly increased, compared with the control group. When HDF was incubated with CM extracts, the protective effect of the samples was found. Treatment with HZCM-2, HZCM-3, LYCM-1, LYCM-2, LYCM-3, and LYCM-4 extracts could significantly decrease the contents of MMP-1 at the concentrations of 25, 50, and 100 µg/mL (Figure 4A). Treatment with HZCM-1 and HZCM-4 extracts could also significantly decrease the contents of MMP-1 at the concentrations of 50 and 100 µg/mL. Moreover, HZCM-3, HZCM-4, LYCM-1, and LYCM-3 extracts could significantly downregulate the relative expression of MMP-1 mRNA at the concentrations of 50 and 100 µg/mL. The relative expression of MMP-1 mRNA was significantly downregulated by HZCM-1, HZCM-2, LYCM-2, and LYCM-4 extracts at the concentrations of 25, 50, and 100 µg/mL (Figure 4B).

The content of PCOL-I in the model group significantly decreased after UVB irradiation, compared with the control group. Treatment with HZCM-2 extract at the concentration of 100 µg/mL could significantly increase the content of PCOL-I. The other extracts could not significantly affect the content of PCOL-I (Figure 4C). The relative expression of PCOL-I mRNA was significantly downregulated after UVB irradiation, compared with the control group. Treatment with HZCM-2 and HZCM-3 extracts at the concentration of 100 µg/mL could significantly increase the relative expression of PCOL-I mRNA.

Skin is easily affected by various external factors, which could form wrinkles and irregular pigmentation and accelerate abnormal skin aging [32]. Among the external factors, UV irradiation is an important factor, which cause DNA damage and the production of ROS, resulting in the change of gene expression level, the decrease of extracellular matrix protein in content, the collapse of skin structure, and the generation of skin wrinkles [33,34]. It was clarified that chronic UV irradiation could induce MMPs expression and repress collagen synthesis [35]. The MMPs, a family of Ca^2+^-dependent, Zn^2+^-containing endopeptidases, could break down most proteins within the extracellular matrix [36]. MMP-1 could cleave all types of collagen and reduce the secretion of TGF-β1, which is a key cytokine involved in the synthesis of PCOL-I [37]. MMP-1 and PCOL-I were important biomarkers of skin aging, and the balance of their expression levels is important for extracellular matrix structure in the skin. In this study, HZCM-2 extract could significantly reduce the production of MMP-1 and improve the production of PCOL-I, displaying better anti-aging activity.

It is reported that free radicals are associated with several skin problems, such as aging, hyperpigmentation, and inflammation [38]. CM extracts have abundant antioxidants, which could prevent the radical formation and/or scavenge the radicals, resulting in alleviating oxidative damage. The activation of MMPs is associated with free radicals by oxidizing the enzyme catalytic center [39]. The antioxidant and anti-aging effects of CM extracts were clarified in this study, especially the collection period of CM.

### 2.5. Chemical Compositions of CM Extracts

**Figure 5:**
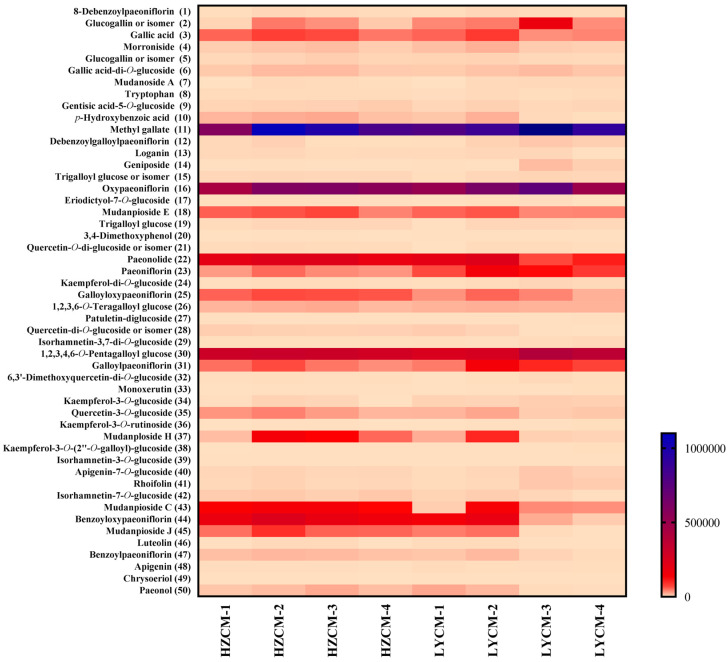
The peak intensities of the Cortex Moutan extracts. The peak intensities of the related compound in the Cortex Moutan (CM) extracts were obtained in UFLC-Q-TOF-MS negative mode. HZCM-1, HZCM-2, HZCM-3, and HZCM-4: CM was collected in Heze in March, June, September, and December, respectively. LYCM-1, LYCM-2, LYCM-3, and LYCM-4: CM was collected in Luoyang in March, June, September, and December, respectively.

Compounds in CM were identified by the UPLC-Q-TOF-MS method through the comparison of retention time, accurate molecular weight, and fragmentography with the reference standards. When the standards were unavailable, compounds were tentatively identified by the accurate molecular weight and fragmentation pathway, which carried out the same kind of compounds as reported in the literature [20]. Eight CM extracts at the concentration of 100 µg/mL were analyzed by the UPLC-Q-TOF-MS method. As shown in Figure 5, a total of 50 compounds, including 17 monoterpenoids, 19 flavonoids, 13 phenols, and 1 amino acid were identified or tentatively identified in the CM extracts.

Monoterpenoids are present as ubiquitous phytochemicals in the CM extracts, which are characterized by two isoprene units and have the molecular formula C_10_H_16_. Among them, morroniside (**4**), loganin (**13**), geniposide (**14**), oxypaeoniflorin (**16**), paeoniflorin (**23**) and benzoyl paeoniflorin (**47**) were precisely identified by comparing with their reference standards, respectively. Moreover, 8-Debenzoylpaeoniflorin (**1**), mudanoside A (**7**), debenzoylgalloylpaeoniflorin (**12**), mudanpioside E (**18**), paeonolide (**22**), galloyloxy paeoniflorin (**25**), galloyl paeoniflorin (**31**), mudanploside H (**37**), mudanpioside C (**43**), benzoyloxypaeoniflorin (**44**), and mudanpioside J (**45**) were tentatively identified as monoterpenoids.

Flavonoids, especially quercetin, kaempferol, isorhamnetin, apigenin, and their derivatives, were other components identified in the CM extracts. Compounds **35**, **41**, **46**, and **48** were precisely identified as quercetin-3-*O*-glucoside, rhoifolin, luteolin, and apigenin, respectively. The fragment behaviors of flavonoids were investigated well in positive and negative ion modes. There were also 15 flavonoids, including eriodictyol-7-*O*-glucoside (**17**), quercetin-*O*-di-glucoside or isomer (**21**), kaempferol-di-*O*-glucoside (**24**), patuletin-diglucoside (**27**), quercetin-di-*O*-glucoside or isomer (**28**), isorhamnetin-3,7-di-*O*-glucoside (**29**), 6,3’-dimethoxyquercetin-di-*O*-glucoside (**32**), monoxerutin (**33**), kaempferol-3-*O*-glucoside (**34**), kaempferol-3-*O*-rutinoside (**36**), kaempferol-3-*O*-(2’’-*O*-galloyl)-glucoside (**38**), isorhamnetin-3-*O*-glucoside (**39**), apigenin-7-*O*-glucoside (**40**), isorhamnetin-7-*O*-glucoside (**42**), and chrysoeriol (**49**), were tentatively identified.

There were 13 phenols and its derivatives found in the CM extracts. Compounds **3**, **10**, **30,** and **50** were directly identified as gallic acid, *p*-hydroxybenzoic acid, 1,2,3,4,6-*O*-pentagalloyl glucose, and paeonol by the comparison of their reference standards, respectively. Compounds **2** and **5** were tentatively identified as glucogallin or isomer. Compounds **15** and **19** were tentatively identified as trigalloyl glucose or isomer. Gallic acid-di-*O*-glucoside (**6**), gentisic acid-5-*O*-glucoside (**9**), methyl gallate (**11**), 3,4-dimethoxyphenol (**20**), 1,2,3,6-*O*-Teragalloyl glucose (**26**), were also tentatively identified as phenolic derivatives.

All 50 compounds could be identified or tentatively identified in the HZCM-2, HZCM-3, LYCM-2, and LYCM-3 extracts. There were 45 compounds found in HZCM-1 and LYCM-1extracts. The extracts of HZCM-1 and LYCM-1 collected in March lacked of mudanoside A (**7**), monoxerutin (**33**) and kaempferol-3-*O*-rutinoside (**36**). Moreover, 3,4-dimethoxyphenol (**20**) and quercetin-di-*O*-glucoside or isomer (**28**) could not be found in HZCM-1, and quercetin-*O*-di-glucoside or isomer (**21**) and 6,3’-dimethoxyquercetin-di-*O*-glucoside (**32**) could not be found in LYCM-1. For the HZCM-4 collected in December, 5 components, 3,4-dimethoxyphenol (**20**), kaempferol-3-*O*-glucoside (**34**), kaempferol-3-*O*-rutinoside (**36**), luteolin (**46**), and chrysoeriol (**49**) were not detected. Eeriodictyol-7-*O*-glucoside (**17**) and quercetin-di-*O*-glucoside or isomer (**28**) could not be found in LYCM-4. Chemical compositions of eight CM extracts were similar based on the results of UPLC-Q-TOF-MS analysis though the CM samples collected from different periods and different producing areas.

### 2.6. Determination of Phytochemicals in CM Extracts

In this study, although there were 50 compounds found by the UFLC-Q-TOF-MS method, it was difficult to quantify all compounds by the HPLC-DAD method due to the trace contents in the extracts and no/weak UV absorption. As the UFLC-Q-TOF-MS results showed, phenols and monoterpenoids were predominant constituents. Four phenols and three monoterpenoids were determined in all CM extracts (Table 2).

In terms of time, for HZCM extracts, the highest content of seven components was found in HZCM-2 or HZCM-3 extracts. Between HZCM-2 and HZCM-3 extracts, the difference of seven components was less than one-fold. In the HZCM samples collected in four periods, the content differences of seven components were less than 1.6-fold. For LYCM extracts, the contents of gallic acid (**3**), *p*-hydroxybenzoic acid (**10**), paeoniflorin (**23**), and benzoyl paeoniflorin (**47)** in LYCM-2 extract were the highest. The contents of oxypaeoniflorin (**16**) and 1,2,3,4,6-*O*-pentagalloyl glucose (**30**) were the highest in LYCM-3 extract. Interestingly, the content of paeonol (**50**) in the LYCM-1 extract was the highest. The contents of gallic acid (**3**), paeoniflorin (**23**), and 1,2,3,4,6-*O*-pentagalloyl glucose (**30**) were relatively stable in the four periods, with less than a one-fold difference. The difference of oxypaeoniflorin (**16**) was about one-fold during the period. The contents of benzoyl paeoniflorin (**47**), *p*-hydroxybenzoic acid (**10**), and paeonol (**50**) had 3.4-, 10.6-, and 46.4-fold differences, respectively. After comparing the contents of seven components in HZCM and LYCM extracts, the difference of the collection period was obvious in terms of chemical contents. For HZCM extracts, the contents of seven components changed little from June to September. The contents of *p*-hydroxybenzoic acid (**10**) and paeonol (**50**) increased by 12.1% and 24.6%, respectively, while the content of paeoniflorin (**23**) decreased by 16.7%. For LYCM extracts, oxypaeoniflorin (**16**) increased slightly from June to September, while the content of 1,2,3,4,6-*O*-pentagalloyl glucose (**30**) increased by 35%. The content of paeoniflorin (**23**) decreased by 12.5%. Gallic acid (**3**), *p*-hydroxybenzoic acid (**10**), benzoyl paeoniflorin (**47**), and paeonol (**50**) decreased exponentially from June to September. There was no significant difference in the contents of seven components between HZCM-2 and HZCM-3 extracts, with the stable peak value, but they were higher than that of HZCM-1 and HZCM-4 extracts. The contents of seven components in the LYCM extracts reached peak values at different periods. There was a variation in the contents of seven compounds among the CM samples, especially LYCM samples.

It was reported that the contents of benzoic acid, gallic acid (**3**), paeoniflorin (**23**), *p*-hydroxybenzoic acid (**10**), and methyl gallate (**11**) were significantly increased, and the genes involved in phenolic metabolism, *PPO*, *DAHPS*, *4CL*, and *SDH*, were differentially expressed after rooting compared with before rooting [40]. Gallic acid (**3**) was one kind of raw material for the synthesis of complex secondary metabolites in the early stage of growth, its content in CM was higher than others in a one-year growth period [41]. The content of paeonol (**50**) increases with the growth of CM. It is considered that paeonol (**50**) is one of the marker components in the growth and maturity of CM. In addition, monoterpenoids also increase until the three-year growth period. Before the four-year growth period, the longer growth period, the quality of CM is better [41]. In this study, three-year old CM samples were used, and monoterpenoids, phenols, and its derivatives were determined, which was consistent with the reported reference. It is reported that oxypaeoniflorin (**16**), paeoniflorin (**23**), and benzoyl paeoniflorin (**47**), as characteristic monoterpenoids were at the contents of 2.05, 7.05, and 3.30 mg/g, respectively [42]. In this study, except for benzoyl paeoniflorin (**47**), the contents of oxypaeoniflorin (**16**) and paeoniflorin (**23**) were much higher than that of other reports. The difference might be caused by the botanical origin and cultured and collected conditions.

### 2.7. Pearson’s Correlation Analysis between Phytochemicals and Bioactivities

**Figure 6:**
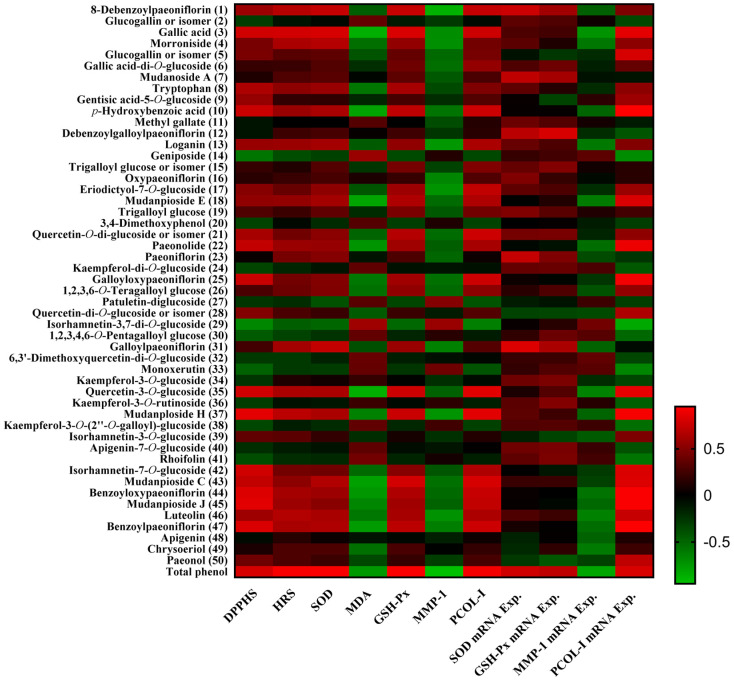
Correlation analysis between phytochemicals and bioactivities. The numbered compounds are consistent with that in Figure 5. DPPHS: DPPH scavenging; HRS: hydroxyl radical scavenging.

Pearson’s correlation analysis was performed to understand the relationship between the phytochemicals and their bioactivities [43]. The correlation coefficient (r) of a single component to each bioactivity is shown in Figure 6.

TPC had a significant positive correlation with DPPH (r = 0.800, *p* < 0.05) and hydroxyl radical (r = 0.924, *p* < 0.01) scavenging in vitro. In ex vivo experiment, TPC had a significant positive correlation with the activities of SOD (r = 0.935, *p* < 0.01) and GSH-Px (r = 0.931, *p* < 0.01), and had a significant negative correlation with the content of MDA (r = −0.754, *p* < 0.05) and MMP-1(r = −0.946, *p* < 0.01). Moreover, TPC had a significant positive correlation with the relative expressions of SOD (r = 0.769, *p* < 0.05) and GSH-Px (r = 0.713, *p* < 0.05) mRNA, and had a significant negative correlation with the relative expression of MMP-1 (r = −0.819, *p* < 0.05) mRNA. TPC had a significant positive correlation with the content of PCOL-I (r = 0.910, *p* < 0.01) and the relative expression of PCOL-I (r = 0.810, *p* < 0.05) mRNA.

The correlation of phytochemicals and the antioxidant activity were further investigated. DPPH radical scavenging had a significantly positive significant correlation with gallic acid (**3**), *p*-hydroxybenzoic acid (**10**), paeonolide (**22**), galloyloxy paeoniflorin (**25**), quercetin-3-O-glucoside (**35**), mudanploside H (**37**), isorhamnetin-7-*O*-glucoside (**42**), mudanpioside C (**43**), benzoyloxypaeoniflorin (**44**), mudanpioside J (**45**), and benzoyl paeoniflorin (**47**). Hydroxyl radical scavenging had a significantly positive correlation with gallic acid (**3**). SOD activity had a significantly positive correlation with gallic acid (**3**) and galloyl paeoniflorin (**31**). GSH-Px activity had a significantly positive correlation with gallic acid (**3**), quercetin-3-*O*-glucoside (**35**), mudanploside H (**37**), and mudanpioside C (**43**). The content of MDA had a significant negative correlation with gallic acid (**3**), *p*-hydroxybenzoic acid (**10**), mudanpioside E (**18**), paeonolide (**22**), quercetin-3-*O*-glucoside (**35**), mudanpioside C (**43**), benzoyloxypaeoniflorin (**44**), and benzoyl paeoniflorin (**47**). SOD mRNA relative expression had a significantly positive correlation with mudanoside A (**7**), paeoniflorin (**23**) and galloyl paeoniflorin (**31**). The relative expression of GSH-Px mRNA had a significantly positive correlation with debenzoylgalloylpaeoniflorin (**12**). Gallic acid (**3**) and its derivatives were strong natural antioxidants and could scavenge free radicals [44,45]. Galloyloxypaeoniflorin (**25**), benzoyloxypaeoniflorin (**44**) had DPPH radical scavenging activity [46,47]. Paeoniflorin (**23**) could increase the activity of SOD [48]. The antioxidant activity is measured by the basic structural organization of the compounds. It is reported that the substituents on the phenyl ring and the conjugated carbon skeleton play an important role in the antioxidant property of the compounds [49].

The content of MMP-1 had a significant negative correlation with morroniside (**4**), loganin (**13**), eriodictyol-7-*O*-glucoside (**17**), mudanploside H (**37**), and luteolin (**46**), and its relative expression had a significant negative correlation with gallic acid (**3**). The content of PCOL-I and its mRNA relative expression had a significantly positive correlation with gallic acid (**3**), *p*-hydroxybenzoic acid (**10**), galloyloxy paeoniflorin (**25**), quercetin-3-*O*-glucoside (**35**), mudanploside H (**37**), mudanpioside C (**43**), benzoyloxypaeoniflorin (**44**), mudanpioside J (**45**), and benzoyl paeoniflorin (**47**). Additionally, the content of PCOL-I had a significantly positive correlation with 8-debenzoylpaeoniflorin (**1**), eriodictyol-7-*O*-glucoside (**17**), and quercetin-*O*-di-glucoside or isomer (**21**). The relative expression of PCOL-I mRNA had a significantly positive correlation with glucogallin or isomer (**5**), mudanpioside E (**18**), paeonolide (**22**), isorhamnetin-7-*O*-glucoside (**42**), and luteolin (**46**), and had a significant negative correlation with isorhamnetin-3,7-di-*O*-glucoside (**29**). Gallic acid (**3**), mudanploside H (**37**), and luteolin (**46**) could affect the productions of MMP-1 and PCOL-I in HDF. The iridoid glycoside fraction containing morroniside (**4**) and loganin (**13**) could downregulate the expression of MMP-1 mRNA in UVB irradiated HS68 cells [50]. It is found that luteolin (**46**) could reduce the production of MMP-1 in SW982 cells [51]. Gallic acid (**3**) could decrease the production of MMP-1 and increase PCOL-I secretion in UVB irradiated HDF cells [52]. Quercetin-3-*O*-glucoside (**35**) could downregulate the mRNA expression of MMP-1 while upregulate the mRNA expression of PCOL-I in UVB irradiated HDF [53].

Plant metabolomics is to study the small molecule metabolites of plants of different species, different gene types, or different ecological types in different growth stages, or before and after certain stimulation. Generally, it involves carrying out quantitative and qualitative analysis, and finding out the change of metabolic principle. A previous study reported that the UPLC-QTOF/MS-based metabolomic analysis combined with the multivariate statistical analysis could provide six potential stage-dependent and bioactive markers—gallic acid, methyl gallate, geniposide, trigalloyl glucose, paeoniflorin and diosmin, which are important criteria for evaluating the quality of *P. suffruticosa* flowers [20]. Multivariate statistical analysis is concerned with data that consist of sets of measurements on a number of individuals or objects. In this study, for CM extract from different periods, there were 25 compounds significantly affecting the in vitro and ex vivo antioxidant activities and the relative expression of mRNA. Among the critical compounds, monoterpenoids, and its derivatives, a total of 15 compounds were predominant phytochemicals, followed by 6 flavonoids and 4 phenols. The results suggest that, in this study, the critical compounds found in CM extracts exhibited biological activities against oxidative stress and aging. Moreover, gallic acid (**3**) was the most important compound, which contributed to antioxidant and anti-aging activities. *p*-Hydroxybenzoic acid (**10**), quercetin-3-*O*-glucoside (**35**), mudanpioside C (**43**), benzoyloxypaeoniflorin (**44**), and benzoyl paeoniflorin (**47**) were noticeable according to the results of the Pearson’s correlation analysis. The six compounds should be the biomarkers in the product development and the quality control of CM (where it was used for external usage).

## 3. Materials and Methods

### 3.1. Plant Materials

CM samples, root bark of 3-year-old *P. suffruticosa* from Heze, Shandong province, China, were collected on March 30 (HZCM-1, voucher no.: 2018SPS03011), June 30 (HZCM-2N, voucher no.: 2018SPS06023), September 30 (HZCM-3, voucher no.: 2018SPS09017), and December 30 (HZCM-4, voucher no.: 2018SPS12009), 2018, respectively. CM samples, root bark of 3-year-old *P. suffruticosa* from Luoyang, Henan province, China, were collected On March 30 (LYCM-1, voucher no.: 2018SPS03012), June 30 (LYCM-2, voucher no.: 2018SPS06024), September 30 (LYCM-3, voucher no.: 2018SPS09018), and December 30 (LYCM-4, voucher no.: 2018SPS12010), 2018, respectively. All voucher specimens were stored at Southern Medical University, Guangdong, China.

### 3.2. Chemicals and Reagents

Paeoniflorin, oxypaeoniflorin, benzoyl paeoniflorin, paeonol, 1,2,3,4,6-*O*-pentagalloyl glucose, luteolin, and apigenin were purchased from Chengdu Pufei De Biotech Co., Ltd. (Chengdu, China). Gallic acid, tryptophan, and *p*-hydroxybenzoic acid were purchased from the National Institute for the Control of Pharmaceutical and Biological Products (Beijing, China). Morroniside, loganin, and geniposide were purchased from Chengdu Biopurify Phytochemicals, Ltd. (Chengdu, China). Quercetin-3-*O*-glucoside and rhoifolin were purchased from Sigma-Aldrich (Shanghai, China). Moreover, 2,4,6-Tris(2-pyridyl)-1,3,5-triazine was purchased from Shanghai Yuanye Biotechnology Co., Ltd. (Shanghai, China). The 2,2-Diphenyl-1-picrylhydrazyl (DPPH), butylated hydroxytoluene (BHT), β-carotene, and vitamin C (VC) were purchased from Shanghai Ekear Bio-Tech Co., Ltd. (Shanghai, China). Dulbecco’s Modified Eagle Medium (DMEM) and fetal bovine serum (FBS) were purchased from Gibco (Thermo Scientific, Waltham, MA, USA). HPLC-grade acetonitrile and LC/MS grade methanol were purchased from Fisher Scientific (Fair Lawn, NJ, USA). Water was obtained from an ultrapure water system (Purelab Plus, Pall, Port Washington, NY, USA).

### 3.3. Sample Preparation

The dried and ground CM sample (100 g) was extracted with 50% (*v*/*v*) ethanol (1000 mL) in a KQ600DE ultrasonic bath (Kunshan, China) for 40 min at room temperature. The repeated extraction was performed after filtration. The combined filtrate was evaporated under vacuum to yield the 50% ethanolic extract. The dried 50% ethanolic extract was weighed accurately and dissolved in 50% ethanol to obtain a series of solutions with different concentrations for the further study.

### 3.4. Total Phenolic Content Assay

Total phenolic content (TPC) was measured according to the described method [54]. Sample solution (1.0 mL) was mixed with the Folin–Ciocalteu reagent (5.0 mL; 0.1 mmol/L) and then incubated at room temperature for 5 min. After, a sodium carbonate solution (4.0 mL; 75 g/L) was added, and the mixture was incubated at room temperature for 30 min in darkness. A gallic acid solution was used as the standard and the absorbance was measured at 765 nm by the spectrophotometer (UV-6100; Shanghai Metash Instrument Co., Ltd., Shanghai, China). TPC was expressed as mg gallic acid equivalents per gram of extract (mg GAE/g extract). All measurements were performed in triplicate.

### 3.5. Antioxidant Assay

#### 3.5.1. DPPH Radical Scavenging Activity Assay

DPPH radical scavenging activity was measured according to the described method [55]. Briefly, a sample solution (1.0 mL) was mixed with a freshly prepared methanol solution of DPPH (1.0 mL; 80 μg/mL), and then incubated for 30 min at room temperature. The absorbance was measured at 517 nm by spectrophotometer. VC was used as a standard antioxidant compound. All measurements were performed in triplicate. In the control experiment, 50% ethanol (*v*/*v*, 2.0 mL) was used. The radical scavenging capability of DPPH was calculated using the following equation: scavenging activity (%) = (1 − A_sample_/A_control_) × 100, where A_sample_ and A_control_ are the absorbance of the sample and the control, respectively.

#### 3.5.2. Hydroxyl Radical Scavenging Activity Assay

The hydroxyl radical scavenging activity was measured according to the described method [54]. The hydroxyl radical was produced by the Fenton reaction between ferrous sulfate and hydrogen peroxide. Briefly, a sample solution (2.0 mL), a ferrous sulfate solution (2.0 mL; 5.0 mmol/L), a salicylic acid solution (2.0 mL; 5.0 mmol/L), and a hydrogen peroxide solution (2.0 mL; 5.0 mmol/L) were added to the test tubes, and then the mixture was incubated at 37 °C for 1 h. The absorbance was measured at 510 nm by spectrophotometer. VC was used as a standard antioxidant compound. In the control experiment, 50% ethanol (*v*/*v*, 2.0 mL) was used. All measurements were performed in triplicate. The hydroxyl radical scavenging capability was calculated using the following equation: scavenging activity (%) = ([1 − (A_sample_ − A_-hydrogen peroxide_)/A_control_) × 100, where A_sample_, A_-hydrogen peroxide_, and A_control_ are the absorbance of the sample, the sample without hydrogen peroxide, and the control, respectively.

#### 3.5.3. Ferric Reducing Antioxidant Power (FRAP) Assay

In this study, FRAP assays were performed according to the described method [55]. The antioxidants could restore ferric tripyridyl triazine (Fe^3+^-TPTZ) to blue Fe^2+^-TPTZ under acidic conditions. A FRAP solution was prepared by mixing acetate buffer (300 mM, pH 3.6), TPTZ (10 mM), and FeCl_3_ solution (20 mM) in a ratio of 10:1:1 (*v*/*v*/*v*). A sample solution (0.15 mL) was mixed with the FRAP solution (2.85 mL) and incubated at 37 °C for 30 min. The total antioxidant activity of the samples could be measured by the absorbance of Fe^2+^-TPTZ at 593 nm. The results of the FRAP assay were reported in mM FeSO_4_. All measurements were performed in triplicate.

#### 3.5.4. Inhibition of β-Carotene Bleaching Assay

Inhibitory ability of β-carotene bleaching was measured according to the described method [55]. Briefly, a β-carotene solution was prepared by dissolving 2.0 mg in chloroform (10 mL). A solution (2.0 mL) was added into a round-bottom flask. After the chloroform was removed at 40 °C under vacuum, linoleic acid (40 mg), Tween-80 emulsifier (400 mg), and distilled water (100 mL) were added to the flask with vigorous shaking. An aliquot (4.8 mL) of this emulsion was transferred to different test tubes containing the sample solution (0.2 mL). A portion of the emulsion (4.8 mL) combined with 50% ethanol (0.2 mL) was used as a control. The tubes were shaken and incubated at 50 °C in a water bath. As soon as the emulsion was added, the absorbance was measured at 470 nm at 0 min and at 15-min intervals over a 120 min period. All measurements were performed in triplicate. The β-carotene bleaching inhibition was calculated using the following equation: bleaching inhibition (%) = (1 − (A_sample_-0 − A_sample-t)_/(A_control-0_ − A_control-t_)) × 100, where A_sample-0_ and Ac_ontrol-0_ are the initial absorbance (t = 0 min) of the sample and the control, respectively; A_sample-t_ and A_control-t_ are the absorbance (t = 15, 30, 45, 60, 75, 90, 105, and 120 min) of the sample and the control, respectively.

### 3.6. Cell Culture

Hs68 HDF purchased from American Type Culture Collection (ATCC, Manassas, VA, USA) were maintained in complete DMEM with 10% heat-inactivated FBS and 1% penicillin–streptomycin at 37 °C in a humidified incubator containing 5% CO_2_. The subculture was conducted at a split ratio of 1:5, when cells were above 80% confluence.

### 3.7. Cell Viability

The cytotoxicity of the CM extracts on HDF was measured using the CCK-8 assay. After incubated for 24 h in 96-well plates at a density of 1 × 10^4^ cells/well, the cells were treated with serial concentrations of samples (0, 25, 50, and 100 μg/mL) in triplicate, and then incubated with complete medium for 24 h. Finally, 10 μL of CCK-8 was added to each plate and incubated at 37 °C for 1 h. The absorption wavelength was measured at 450 nm using a Tecan microplate reader (Tecan Group Ltd., Männedorf, Switzerland). The cell viability was evaluated by comparing the absorbance values between the treatment groups and the control group, which was considered as 100%.

### 3.8. UVB Irradiation and Sample Treatment

HDF cells were incubated for 24 h in 96-well plates at a density of 1 × 10^4^ cells/well, and then were treated with serial concentrations (25, 50, and 100 μg/mL) of CM extracts in serum-free medium for 1 h. After the culture medium was replaced with PBS, the cells were exposed to UVB irradiation (60 mJ/cm^2^) using GL20SE UV lamps (Sankyo Denki, Marine, Japan). The control groups were kept under the same culture conditions without UVB irradiation [56]. The cells were further incubated with complete medium for 24 h, and subjected to different biochemical analyses.

### 3.9. Measurement of SOD, GSH-Px, MDA, MMP-1, and PCOL-I

The activity of superoxide dismutase (SOD) was measured by the available commercial kit according to the manufacturer’s instructions (Nanjing Jiancheng Bioengineering Institute, Nanjing, China). The content of malondialdehyde (MDA) and the activity of glutathione peroxidase (GSH-Px) were measured by colorimetric assay kits, and the levels of MMP-1 and PCOL-I were measured by enzyme-linked immunosorbent assay (ELISA) kits, according to the manufacturer’s instructions (Elabscience Biotechnology Co., Ltd., Wuhan, China).

### 3.10. Real-Time PCR Assay

Total RNA was extracted from the HDF using the RNAprep Pure Cell kit (Qiagen, Valencia, CA, USA) according to the manufacturer’s protocol. RNA concentration and purity were measured by the ratio of 260/280 nm absorbance. The total RNA solution was stored at –80 °C for reverse transcription. The total RNA was converted into the first-strand complementary DNA (cDNA) with a reverse transcription system as follows: 4 μL 5x prime Script RT Master MIX (Takara Bio INC., Kusatsu, Japan), 0.5 μg total RNA, and RNase-free water in a 20-μL reaction. The cDNA was used for RT-PCR and the reaction system contained 10 μL SYBR Premix EX Taq (2×) (Promega Corporation, Madison, USA), 1 μL forward primer (10 μM), 1 μL reverse primer (Table 3) [57,58], and 8 μL cDNA, which was amplified in an Applied Biosystems 7500 Fast Real-time PCR System version v2.3 (Thermo Fisher Scientific, Waltham, MA, USA) under the following reaction conditions: 50.0 °C for 3 min and 95.0 °C for 3 min, followed by 40 cycles of 95.0 °C for 10 s and 60.0 °C for 30 s. The threshold cycle (C_t_) was analyzed by the instrument’s software; fold changes in the mRNA expression were calculated according to the comparative Ct method (2^−^^△△CT^).

### 3.11. UFLC-Q-TOF-MS Analysis

#### 3.11.1. System and Conditions

Ultra-fast liquid chromatography (UFLC) analysis was performed using a Shimadzu UFLC XR system (Shimadzu Corp., Kyoto, Japan) and an Agilent Eclipse Plus C18 column (2.1 i.d. ×100 mm, 1.8 μm, Agilent Technologies, CA, USA). The mobile phase was composed of methanol (A) and 0.1% (*v*/*v*) formic acid solution (B) using a linear gradient elution of 5–100% A within 30 min at 0.3 mL/min. The injection volume was 5 μL, and the column temperature was set at 25 °C. The identification experiment was performed using AB SCIEX Triple TOF 5600 plus mass spectrometer system (AB SCIEX, Foster City, CA, USA). The operation system was the Analyst TF 1.6 software (AB SCIEX, Foster City, CA, USA). The parameters of the MS detector were set as follows: ion source gas 155 psi; ion source gas 255 psi; curtain gas 30 psi; source temperature 550 °C; ion spray voltage floating 4500 V; collision energy 35 eV; collision energy spread 15 eV; and declustering potential 80 eV. Spectra were acquired in a scan range from *m*/*z* 100–1500. Both positive and negative ion modes were used for compounds ionization. Nitrogen was used as the nebulizer and auxiliary gas.

#### 3.11.2. Establishment of Tentative Peak Assignment

The UFLC-Q-TOF-MS data of CM samples were extracted and analyzed using the PeakView software (AB SCIEX, Foster City, CA, USA) and the XIC manager tool, which provided the quasi-molecular weights, mass errors, and isotope pattern fits. The predicted formula with errors less than ±5 ppm was searched against the compounds reported in the genus Paeonia to obtain the tentative identification. The identified compounds within the CM extracts were further confirmed by determining the possible elemental compositions of the fragment ions and the proposed fragmentation pathways using their MS spectrum [27].

### 3.12. HPLC-DAD Analysis

High performance liquid chromatography (HPLC) analysis was performed using an Agilent (Foster City, CA, USA) 1290 UPLC-DAD system and a reverse-phase Agilent EC-C18 column (3.0 i.d. × 100 mm, 2.7 µm, Agilent Technologies, Foster City, CA, USA). A gradient elution mobile phase system composed of 0.5% (*v*/*v*) formic acid solution (A) and acetonitrile (B) was applied as follows: 0–3 min, 5–8% B; 3–4 min, 8–13% B; 4–13 min, 13–14% B; 13–14 min, 14–20% B; 14–29 min, 20–30% B; 29–30 min, 30–50% B; 30–33 min, 50–95% B. The mobile phase flow rate was 0.3 mL/min. The column temperature was maintained at 30 °C. The injection volume was 5 μL and the UV wavelength was set at 254 nm [27].

### 3.13. Statistical Analysis

The results were expressed as mean ± standard deviation (SD) of each experiment. The obtained data were analyzed using a one-way analysis of variance (ANOVA) followed by Tukey’s multiple comparison test. A *p* value < 0.05 was considered significant. Pearson’s correlation analysis was performed to characterize the correlation between the phytochemicals and the bioactivities. One-way ANOVA and Pearson’s correlation analysis were performed using the software IBM SPSS Statistics (version 19.0).

## 4. Conclusions

In this study, it was found that CM had different antioxidant and anti-aging properties at different collection periods and different producing areas. A total of 50 compounds, including 17 monoterpenoids, 19 flavonoids, 13 phenols, and 1 amino acid were identified or tentatively identified by the UFLC-Q-TOF-MS method. The content differences of seven compounds, including four phenols and three monoterpenoids was found in CM samples from different collection periods and different producing areas, according to the determined results. Meanwhile, the results of multivariate statistical analysis suggested that the multiple components in CM contributed to their antioxidant and anti-aging activities and six critical compounds as biomarkers should be noticeable. To have excellent antioxidant and anti-aging activities for external use, and to contain the high levels of bioactive compounds, the optimal time to collect CM was from April to September.

## Figures and Tables

**Table 1:** Extraction yields, total phenolic contents, and antioxidant activities (IC_50_ values) of the Cortex Moutan extracts.

Samples	Extraction Yields (%)	Total Phenolic Content (mg GAE/g ext.)	DPPH Radical Scavenging Activity (μg/mL)	Hydroxyl Radical Scavenging Activity (mg/mL)
VC	–	–	9.81 ± 0.48	0.11 ± 0.004
HZCM-1	20.33 ± 0.99	73.47 ± 1.01	88.00 ± 2.37	0.48 ± 0.02
HZCM-2	21.73 ± 1.72	112.95 ± 3.97	42.70 ± 0.79	0.20 ± 0.006
HZCM-3	22.21 ± 0.84	80.80 ± 4.48	79.98 ± 1.98	0.54 ± 0.02
HZCM-4	21.44 ± 1.75	64.36 ± 2.85	68.47 ± 2.55	0.55 ± 0.02
LYCM-1	21.13 ± 0.93	67.71 ± 2.09	112.74 ± 0.66	0.48 ± 0.04
LYCM-2	22.53 ± 1.04	107.52 ± 3.03	40.38 ± 1.86	0.19 ± 0.01
LYCM-3	21.67 ± 1.07	80.70 ± 2.95	85.45 ± 0.80	0.53 ± 0.03
LYCM-4	22.01 ± 0.92	63.81 ± 3.96	138.36 ± 2.78	0.69 ± 0.04

Vitamin C: VC; HZCM-1, HZCM-2, HZCM-3 and HZCM-4: Cortex Moutan (CM) collected in Heze in March, June, September, and December, respectively. LYCM-1, LYCM-2, LYCM-3, and LYCM-4: CM collected in Luoyang in March, June, September, and December, respectively. Data are expressed as mean ± SD (*n* = 3).

**Table 2:** Contents of four phenols and three monoterpenoids in the Cortex Moutan extracts determined using HPLC-DAD analysis.

Compounds	Contents (mg/g Extracts)							
	HZCM-1	HZCM-2	HZCM-3	HZCM-4	LYCM-1	LYCM-2	LYCM-3	LYCM-4
Gallic acid (**3**)	11.39 ± 0.86	14.86 ± 1.12	14.51 ± 0.75	9.33 ± 0.71	10.77 ± 1.30	16.55 ± 1.05	8.40 ± 1.10	9.27 ± 0.95
*p*-Hydroxybenzoic acid (**10**)	2.99 ± 0.39	5.29 ± 0.34	5.93 ± 0.27	2.36 ± 0.23	2.91 ± 0.19	4.64 ± 0.42	0.73 ± 0.12	0.40 ± 0.04
Oxypaeoniflorin (**16**)	5.95 ± 0.47	8.31 ± 0.54	8.24 ± 0.64	6.23 ± 0.38	5.38 ± 0.28	9.79 ± 0.41	10.25 ± 0.35	5.11 ± 0.12
Paeoniflorin (**23**)	9.41 ± 1.07	14.23 ± 0.88	11.85 ± 0.55	10.80 ± 0.70	16.69 ± 0.77	21.01 ± 1.48	18.38 ± 0.71	19.50 ± 0.72
1,2,3,4,6-*O*-Pentagalloyl glucose (**30**)	30.02 ± 1.26	34.00 ± 1.60	34.87 ± 1.40	26.44 ± 1.06	23.49 ± 2.03	28.09 ± 1.04	38.01 ± 1.66	35.49 ± 1.46
Benzoyl paeoniflorin (**47**)	0.47 ± 0.04	0.49 ± 0.02	0.49 ± 0.04	0.42 ± 0.02	0.35 ± 0.04	0.57 ± 0.05	0.24 ± 0.03	0.13 ± 0.02
Paeonol (**50**)	13.21 ± 1.39	18.11 ± 1.36	22.58 ± 1.63	16.93 ± 0.55	22.77 ± 2.25	18.13 ± 1.34	0.52 ± 0.04	0.48 ± 0.03

HZCM-1, HZCM-2, HZCM-3 and HZCM-4: Cortex Moutan (CM) collected in Heze in March, June, September, and December, respectively. LYCM-1, LYCM-2, LYCM-3, and LYCM-4: CM collected in Luoyang in March, June, September, and December, respectively. Data are expressed as mean ± SD (*n* = 3).

**Table 3:** Primer sequences for real-time PCR amplification.

Gene Names	Forward Primer Sequences (5′–3′)	Reverse Primer Sequences (5′–3′)
SOD	TGGAGATAATACAGCAGGCT	AGTCACATTGCCCAAGTCTC
GSH-Px	AGAAGTGCGAGGTGAACGGT	CCCACCAGGAACTTCTCAAA
MMP-1	ATTCTACTGATATCGGGGCTTTGA	ATGTCCTTGGGGTATCCGTGTAG
COL1A1	AGGGCCACGAAGACATC	AGATGACGTCATCGCACAACA
GAPDH	ACCACAGTCCATGCCATCAC	CCACCACCCTGTTGCTGTAG

## Sample Availability

Samples of the compounds are not available from the authors.
